# Transcranial pulse stimulation in Alzheimer's disease

**DOI:** 10.1111/cns.14372

**Published:** 2023-07-20

**Authors:** Xinxin Chen, Jiuhong You, Hui Ma, Mei Zhou, Cheng Huang

**Affiliations:** ^1^ Department of Rehabilitation Medicine West China Hospital Sichuan University Chengdu Sichuan China; ^2^ Key Laboratory of Rehabilitation Medicine in Sichuan Province West China Hospital Sichuan University Chengdu Sichuan China; ^3^ School of Rehabilitation Sciences West China School of Medicine Sichuan University Chengdu Sichuan China

**Keywords:** Alzheimer's disease, effect, systematic review, transcranial pulse stimulation

## Abstract

**Background:**

Transcranial pulse stimulation (TPS) is a novel noninvasive ultrasonic brain stimulation that can increase cortical and corticospinal excitability, induce neuroplasticity, and increase functional connectivity within the brain. Several trials have confirmed its potential in treating Alzheimer's disease (AD).

**Objective:**

To investigate the effect and safety of TPS on AD.

**Design:**

A systematic review.

**Methods:**

PubMed, Embase via Ovid, Web of Science, Cochrane Library, CNKI (China National Knowledge Infrastructure), VIP (China Science and Technology Journal Database), and WanFang were searched from inception to April 1, 2023. Study selection, data extraction, and quality evaluation of the studies were conducted by two reviewers independently, with any controversy resolved by consensus. The Methodological Index for Nonrandomized Studies was used to assess the risk of bias.

**Results:**

Five studies were included in this review, with a total of 99 patients with AD. For cognitive performance, TPS significantly improved the scores of the CERAD (Consortium to Establish a Registry for Alzheimer's Disease) test battery, Alzheimer's Disease Assessment Scale (cognitive), Montreal Cognitive Assessment, and Mini‐Mental Status Examination. For depressive symptoms, TPS significantly reduced the scores of the Alzheimer's Disease Assessment Scale (affective), Geriatric Depression Score, and Beck Depression Inventory. By functional magnetic resonance imaging, studies have shown that TPS improved cognitive performance in AD patients by increasing functional connectivity in the hippocampus, parahippocampal cortex, precuneus, and parietal cortex, and activating cortical activity in the bilateral hippocampus. TPS alleviated depressive symptoms in AD patients by decreasing functional connectivity between the ventromedial network (left frontal orbital cortex) and the salience network (right anterior insula). Adverse events in this review, including headache, worsening mood, jaw pain, nausea, and drowsiness, were reversible and lasted no longer than 1 day. No serious adverse events or complications were observed.

**Conclusions:**

TPS is promising in improving cognitive performance and reducing depressive symptoms in patients with AD. TPS may be a safe adjunct therapy in the treatment of AD. However, these findings lacked a sham control and were limited by the small sample size of the included studies. Further research may be needed to better explore the potential of TPS.

**Patient and Public Involvement:**

Patients and the public were not involved in this study.

## Graphical abstract

TPS is promising in improving cognitive performance and reducing depressive symptoms in patients with AD. TPS may be a safe adjunct therapy in the treatment of AD. Fundamental research is needed for the exact mechanism of TPS for AD.

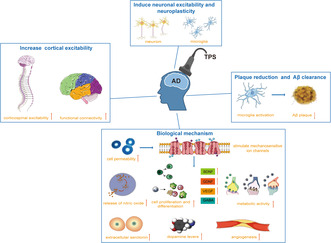

## 1 INTRODUCTION

Approximately 50 million people living with dementia worldwide in 2018, and this number is expected to triple (approximately 139 million) by 2050.^1^ Alzheimer's disease (AD) is a chronic neurodegenerative disease that develops slowly and worsens over time.^2^ According to the World Health Organization, AD is the leading cause of dementia, accounting for about 60%–70% of all cases.^3^ It is characterized by progressive memory loss and cognitive dysfunction and has become one of the most expensive and burdening diseases.^4^ The typical pathological manifestations of AD are senile plaques formed by extracellular aggregation of amyloid beta (Aβ) plaques in the cerebral cortex and limbic regions, and intracellular neurofibrillary tangles caused by hyperphosphorylation of tau protein.^5^ Due to the complex pathogenesis, there is still a lack of effective therapeutic methods to cure or reverse the pathological process of AD.^6^ Cholinesterase inhibitors, n‐methyl‐D‐aspartate receptor partial antagonists, are commonly used in the clinical treatment of AD.^7^ However, these pharmacotherapies are accompanied by adverse effects and have limited effectiveness.^8^ Many studies of AD‐related drugs fail to achieve the intended goal of delaying AD progression in large population clinical trials.^9^ Therefore, there is an urgent need for new alternative nonpharmaceutical approaches, in tandem with medications and smart lifestyle adjustments, to slow, prevent, or cure AD.^9^

In recent years, growing evidence has suggested that noninvasive brain stimulation (NIBS) techniques may have positive effects on cognitive performance in AD.^10^, ^11^, ^12^, ^13^ Common NIBS techniques include transcranial magnetic stimulation (TMS) and transcranial direct current stimulation (tDCS), which are based on electromagnetic effects on the brain. Thus, the spatial resolution is limited due to conductivity effects, which affect not only the actual stimulation site but also other brain regions.^14^ Moreover, TMS and tDCS cannot effectively target neural tissue below the cortical surface because of poor spatial resolution, suffering from deep focality tradeoffs, and experiencing significant attenuation at depth.^15^ In contrast, ultrasonic stimulation can be precisely targeted with a neuronavigation device in such cases, because ultrasound does not rely on electrical conductivity in the brain.^16^ Transcranial pulse stimulation (TPS) is a novel noninvasive technique for navigated neuromodulation based on single ultrashort high‐intensity ultrasound pulses (~3 μs) repeated every 200–300 ms.^17^, ^18^ Due to the high‐spatial resolution of deep focal lengths, TPS can noninvasively modulate neural targets deep in the human cortex.^19^ TPS uses lower focused ultrasound frequencies that can stimulate a depth of up to 8 cm, reaching deep brain structures such as the thalamus lying at a distance between 5 and 6.5 cm from the scalp.^20^ Modeling studies have found that TPS can alter the electrical activity of cortical and subcortical structures.^21^ Compared with transcranial focused ultrasound stimulation, TPS has the advantage of avoiding tissue heating and standing wave phenomena due to the use of very short pulses without periodic waves or long ultrasonic sequences.^22^, ^23^, ^24^ TPS has obtained clinical certification for AD treatment (Conformité Européene mark).^25^

In 2019, the first pilot study^25^ using TPS to treat AD found improvements in cognitive ability and depressive symptoms in patients, which lasted up to 3 months, and confirmed changes in memory network connectivity after TPS treatment by functional magnetic resonance imaging (fMRI). Subsequently, to verify and explore the efficacy, safety, and mechanism of TPS in the treatment of AD, several clinical trials have been completed and reported.^26^, ^27^, ^28^, ^29^ However, the research emphases of these studies were not entirely consistent, and the quality of the studies was different. To provide an unbiased summary and evaluate the quality of these studies, we conducted a systematic review, which can provide suggestions for further research on the treatment of AD by the new TPS technique.

## 2 METHODS

This study followed the Preferred Reporting Items for Systematic Reviews and Meta‐Analyses (PRISMA) statement.^30^ The PROSPERO trial registration number for this study is CRD42023404284.

### 2.1 Search strategy

Two reviewers conducted a comprehensive literature search to identify all articles involving TPS in the treatment of AD. The search strategy is shown in Appendix S1. PubMed, Embase via Ovid, Web of Science, Cochrane Library, CNKI (China National Knowledge Infrastructure), VIP (China Science and Technology Journal Database), and WanFang were searched from inception to April 1, 2023. The search included MeSH and free text terms such as “transcranial pulse stimulation,” “Alzheimer's disease,” and synonyms.

### 2.2 Inclusion and exclusion criteria

Due to the lack of data on this intervention and the suspected lack of randomized controlled trials (RCTs), we considered including all clinical trials in the systematic review. The inclusion criteria were as follows: (1) clinical trials, (2) articles in the English or Chinese language, (3) articles of AD patients diagnosed by an expert in cognitive neurology according to the International Classification of Diseases 10th revision (F00) and National Institute on Aging criteria, and aged above 18 years, and (4) articles exploring the application of TPS. The criteria for exclusion were as follows: irrelevant studies, reviews, duplicate publications, animal experiments, commentaries, editorial materials, patents, or meeting abstracts.

### 2.3 Study selection and data extraction

The eligibility of each article was independently assessed by two reviewers (XXC and JHY), with any divergence finalized through discussion. Duplicate articles were excluded. By reading the titles and abstracts, irrelevant articles were eliminated, and then the full texts of potentially eligible articles were screened. Two reviewers (HM and MZ) performed data extraction independently, with disagreements resolved by consensus. Finally, the data were recorded in a predesigned form. The extracted data included the study's basic information (title, first author, publication year, country, study design); the participants in the study (number, age, gender); intervention details (protocol, parameters, duration, frequency, sessions, follow‐up); outcome measures; and the data of results.

### 2.4 Outcome definitions

The primary outcome measure was the German version of the CERAD Plus (Consortium to Establish a Registry for Alzheimer's Disease) test battery,^31^ and the secondary outcome measures included the Alzheimer's Disease Assessment Scale (ADAS), Montreal Cognitive Assessment (MoCA), Mini‐Mental Status Examination (MMSE), fMRI, Geriatric Depression Score (GDS), Beck Depression Inventory (BDI), and adverse events.

#### 2.4.1 Neuropsychological evaluation

The CERAD was applied for neuropsychological testing, including naming (Boston Naming Test), word fluency (phonemic and categorical), encoding, recognition, constructional praxis, and constructional recall (Figures Copy and Recall), and recall of verbal material (Word List). The CERAD corrected total score (CTS) is an overall outcome parameter of patients' cognitive status. The CERAD logistic regression score (LR) is an important test for AD‐type dementia. The CERAD principal component analysis (PCA) can separately monitor cognitive components of verbal processing, memory, and visual–spatial processing. In addition, the ADAS total score, ADAS cognitive score, MoCA, and MMSE were used as neuropsychological assessments for the cognitive function of the patients. ADAS‐cognitive is a measure of cognitive performance and has been used widely in AD trials.^32^

#### 2.4.2 fMRI

The fMRI results included resting state data (functional connectivity) and task state data (memory test).

#### 2.4.3 Emotional symptom assessment

Assessment methods included ADAS affective score, GDS, and BDI. ADAS‐affective includes interviews about mood and behavior changes in patients. GDS evaluates symptoms of depression in elderly patients based on the 30‐question long‐form. BDI is a 21‐question multiple‐choice self‐report scale that measures the severity of depression.

#### 2.4.4 Adverse events

Assessment methods included the Numeric Rating Scale (NRS), Visual Analogue Scale evaluation (VAS 0–10), and side effects that were recorded in the articles. NRS is used to assess the extent of side effects. The VAS is used to assess pain and pressure.

### 2.5 Risk of bias assessment

The Methodological Index for Nonrandomized Studies (MINORS)^33^ was used to assess the quality of the nonrandomized controlled trials. The risk of bias in each study was assessed independently by two reviewers (XXC and JHY), with any controversy resolved by consensus. The MINORS checklists include eight items (0–16 scores) for noncomparative studies and 12 items (0–24 scores) for comparative studies. Scores for each item ranged from 0 to 2 (0, not reported; 1, reported but inadequate; 2, reported and adequate). Noncomparative studies scoring > 12 or comparative studies scoring > 19 were considered high quality.

## 3 RESULTS

### 3.1 Study characteristics

**FIGURE 1:**
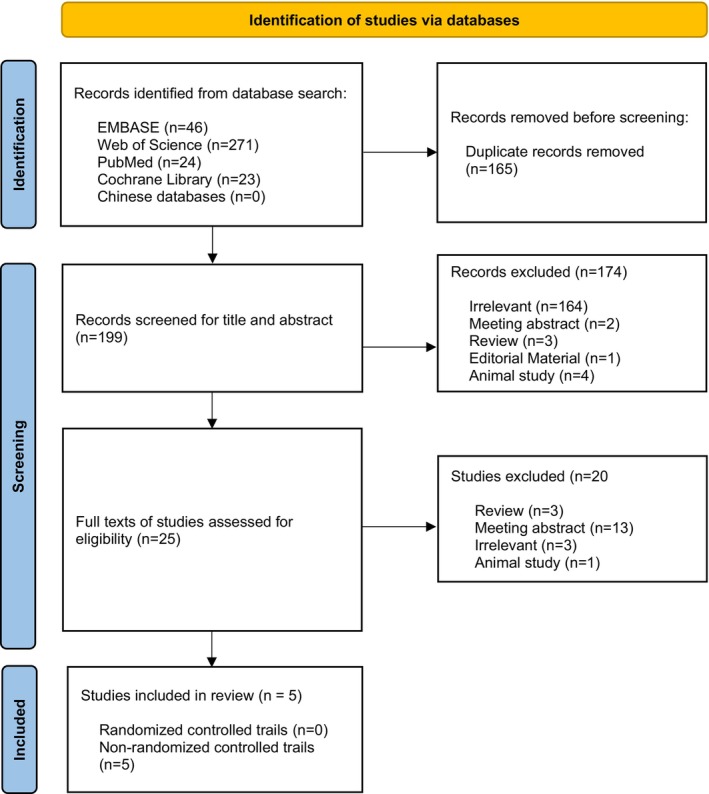
PRISMA flowchart.

From 199 eligible articles, five studies (nonrandomized controlled trials) were included in this review. The process is detailed in the flow diagram in Figure 1. A total of 99 patients with AD were included in this review. The characteristics of the included studies are shown in Table 1.

**TABLE 1:** Characteristics of the included articles.

Author, year, country	Study design	Patients (*N*)	Procedure of TPS	Duration	Location of stimulation	Follow‐up	Outcome measures
Beisteiner, 2019,^25^ Austria	Noncomparative study	35 (center 1 = 19, center 2 = 16)	3 μs, 0.2 mJ/mm^2^ energy flux density, pulse repetition frequency 5 Hz, 6000 pulses per session	2–4 weeks (center 1), 2 weeks (center 2)	Bilateral frontal cortex, bilateral lateral parietal cortex, and extended precuneus cortex (center 1), nonnavigated global cortical stimulation (center 2)	3 months	CERAD total score, CERAD logistic regression score, CERAD principal component analysis, fMRI, GDS, BDI
Cont, 2022,^26^ Germany	Noncomparative study	11	4 Hz, 0.2 mJ/mm^2^ energy flux density, 6000 or 3000 pulses per session	2 weeks (6 sessions) or 2 weeks (12 sessions)	Bilateral frontal cortex, bilateral lateral parietal cortex, extended precuneus cortex, and bilateral temporal cortex	–	ADAS score, MMSE, MoCA, NRS
Dörl, 2022,^27^ Austria	Noncomparative study	18	3 μs, 0.2 mJ/mm^2^ energy flux density, pulse repetition frequency 5 Hz, 6000 pulses per session	2–4 weeks, with three sessions per week	Bilateral frontal cortex, bilateral lateral parietal cortex, and extended precuneus cortex	–	CERAD figural score, fMRI
Matt, 2022,^28^ Austria	Noncomparative study	18	3 μs, 0.2 mJ/mm^2^ energy flux density, pulse repetition frequency 5 Hz, 6000 pulses per session	2–4 weeks, with three sessions per week	Bilateral frontal cortex, bilateral lateral parietal cortex, and extended precuneus cortex	–	BDI, fMRI
Popescu, 2021,^29^ Austria	Noncomparative study	17	3 μs, 0.2 mJ/mm^2^ energy flux density, pulse repetition frequency 5 Hz, 6000 pulses per session	2–4 weeks	Bilateral frontal cortex, bilateral lateral parietal cortex, and extended precuneus cortex	–	CERAD total score, fMRI

Abbreviations: ADAS, Alzheimer's Disease Assessment Scale; BDI, Beck Depression Inventory; CERAD, Consortium to Establish a Registry for Alzheimer's Disease; fMRI, functional magnetic resonance imaging; GDS, Geriatric Depression Score; MMSE, Mini‐Mental Status Examination; MoCA, Montreal Cognitive Assessment; NRS, Numeric Rating Scale;TPS, transcranial pulse stimulation.

### 3.2 Risk of bias assessment

Table 2 shows the risk of bias of the six noncomparative studies. The average MINORS score for the included studies was 9.4. The highest MINORS score of the studies was 12, and no study was considered high quality. Lack of biased assessment of the study endpoint, follow‐up, and study size calculation were the most common reasons for low MINORS scores.

**TABLE 2:** Quality assessment of the included nonrandomized controlled trials using the Methodological Index for Nonrandomized Studies (MINORS).

Assessment	Beisteiner 2019	Cont 2022	Dörl 2022	Matt 2022	Popescu 2021
1. A clearly stated aim	2	2	2	2	2
2. Inclusion of consecutive patients	2	2	2	2	2
3. Prospective collection of data	2	0	2	2	2
4. Endpoints appropriate to the aim of the study	2	2	2	2	2
5. Unbiased assessment of the study endpoint	0	0	0	0	0
6. Follow‐up period appropriate to the aim of the study	2	1	0	0	0
7. Loss to follow up less than 5%	2	0	0	0	0
8. Prospective calculation of the study size	0	0	0	2	2
Total score	12	7	8	10	10

### 3.3 Effect of TPS

#### 3.3.1 Cognitive improvement in AD

Three studies^25^, ^27^, ^29^ reported CERAD test scores, such as CTS, LR, and PCA (memory, verbal, figural), with one study^25^ conducting a three‐month follow‐up indicating the lasting efficacy of TPS in cognitive improvement. Beisteiner et al.^25^ and Popescue et al.^29^ proposed that patients with AD showed a significant pre‐to‐post behavioral improvement in CTS score after TPS intervention. A study by Beisteiner et al.^25^ showed that TPS could ameliorate the LR score and remain stable for more than 3 months. Except for figural capacity, the memory and verbal capacity of AD patients improved significantly after TPS treatment and remained stable for 3 months.^25^ A study by Dörl et al.^27^ also demonstrated a trend in declining visuo‐constructive capabilities (CERAD figural score) and correspondingly global efficiency when the important visuo‐constructive network nodes were omitted during TPS. This decline was compatible with the natural progression of the disease.

Only the study by Cont et al.^26^ reported the ADAS total score, ADAS cognitive score, MMSE, and MoCA. Compared to baseline, TPS significantly increased the ADAS total score and ADAS cognitive score in patients with AD. There was a total improvement in the ADAS total score of 15.76% and in the ADAS cognitive score of 8.65%. However, no significant differences were found in either the MMSE or MoCA. This inconsistency may be due to the small sample size of this study (*n* = 11) and the different sensitivities of different neuropsychological tests. The ADAS is generally more sensitive than the MMSE and MoCA scales.^34^, ^35^, ^36^ Cont et al.^26^ performed a subgroup analysis of patients with AD of varying severity and found that TPS improved more in the group with moderate and severe cognitive impairment than in the group with mild cognitive impairment across all cognitive assessments. This suggests the need to consider a ceiling effect of TPS on mildly affected patients with AD.

#### 3.3.2 fMRI results

Four studies^25^, ^27^, ^28^, ^29^ reported changes in functional connectivity of brain or cortical activation in AD patients pre‐to‐post TPS treatment by fMRI investigations. Furthermore, correlations between functional connectivity and neuropsychological test scores were analyzed. Beisteiner et al.^25^ proposed increased functional connectivity in the hippocampus, parahippocampal cortex, precuneus, and parietal cortex in AD patients after TPS treatment, as well as increased specific activation in the bilateral hippocampus. The increase in functional connectivity values significantly correlated with CERAD scores, suggesting that specific upregulation of memory networks was associated with cognitive ability.^25^ Matt et al.^28^ found that stimulated areas of TPS associated with depression seemed to alleviate depressive symptoms in AD patients by reducing functional connectivity between the ventromedial network (left frontal orbital cortex) and the salience network (right anterior insula). By fMRI, a positive correlation between CERAD figural scores and changes in the global efficiency of the visual construction network was found.^27^ Furthermore, Popescue et al.^29^ indicated a significant correlation between neuropsychological CTS score improvement (pre‐to‐post TPS) and cortical thickness increase in AD's critical default mode network (fMRI data), namely of the left superior parietal lobule and left precuneus. The default mode network, which is important for cognitive and memory performance, is degraded early in the course of AD. Therefore, TPS can regulate the cortical thickness of the stimulated brain region, thereby reducing cortical atrophy in AD.^29^

#### 3.3.3 Improvement of depressive symptoms

Three studies^25^, ^26^, ^28^ reported that TPS reduced depressive symptoms in patients with AD. Beisteiner et al.^25^ indicated that the effects of TIME on the GDS and BDI scores were significant after TPS treatment, with a sustained reduction in depressive symptoms in AD patients for at least 3 months. Matt et al.^28^ demonstrated that TPS stimulation of multiple brain regions, including the extended dorsolateral prefrontal cortex, led to a significant improvement in the BDI. Moreover, Cont et al.^26^ reported a significant improvement in depressive symptoms (ADAS affective score) in AD patients after TPS treatment compared to baseline. Therefore, TPS may be a new adjunct therapy for depression in AD patients.

#### 3.3.4 Adverse events

Only two studies^25^, ^26^ reported the side effects of TPS for AD. Beisteiner et al.^25^ found rare adverse events of painless pressure sensations (17%), pain (8%), headache (4%), and mood deterioration (3%) in their patient population. Cont et al.^26^ proposed that three out of 11 patients (27%) experienced side effects in three out of 75 total sessions (4%). These included pain in the jaw (NRS 4/10), feelings of nausea (NRS 7/10), and drowsiness (NRS 10/10). However, the side effects were reversible and lasted no longer than 1 day. TPS may be safe in the treatment of AD, and large‐sample trials are needed for verification.

## 4 DISCUSSION

To our knowledge, this systematic review is the first to comprehensively evaluate the treatment and safety of TPS in AD. Studies in this review have shown that TPS significantly improved the scores of the CERAD test battery, ADAS‐cognitive, MoCA, and MMSE and significantly reduced the scores of the ADAS‐affective, GDS, and BDI in patients with AD. However, the CERAD figural score (visual construction ability) tended to decline when the important visual construction network nodes were not stimulated during TPS. Studies have also attempted to use fMRI to explore changes in brain functional connectivity or cortical activation in AD patients before and after TPS treatment and their correlations with neuropsychological test scores (CERAD and BDI). The results showed that TPS improved cognitive performance in AD patients by increasing functional connectivity in the hippocampus, parahippocampal cortex, precuneus, and parietal cortex, and activating cortical activity in the bilateral hippocampus. These cortices are closely associated with memory. In additon, TPS alleviated depressive symptoms in AD patients by decreasing functional connectivity between the ventromedial network (left frontal orbital cortex) and the salience network (right anterior insula). The two networks have been proposed as negatively correlated networks for depression.^37^ In addition, TPS can reduce cortical atrophy in AD by increasing cortical thickness in stimulated brain areas. Only one study^25^ conducted a follow‐up measurement and found that TPS continuously improved cognitive capacities and decreased depressive symptoms in AD for at least 3 months.

None of the included studies had a blank or sham control group, so interference by other factors could not be excluded, which may affect the authenticity of the results. The optimal current, frequency, number of pulses, location of stimulation, and sessions for treating AD are unknown, so most studies refer to the research protocols of the first pilot study^25^ for TPS treatment. Most of the included studies used the same TPS procedure, which was 3 μs, 0.2 mJ/mm^2^ energy flux density, pulse repetition frequency 5 Hz, and 6000 pulses per session. The most common duration of TPS treatment was 2 to 4 weeks, three sessions per week. The frequency of the stimulation is one of the important parameters that significantly affect the TPS effect.^38^ Previous studies have found that random frequency parameters (1–5 Hz) increase functional connectivity in the brain compared to nonrandom and spurious stimuli.^39^, ^40^ Morales‐Quezada et al.^41^ also demonstrated that a random frequency of TPS between 6 and 10 Hz also led to an increase in brain functional connectivity, as shown by the facilitation of electroencephalogram spectral power and connectivity measurements. Studies in this review commonly used the bilateral frontal cortex, the bilateral lateral parietal cortex, and the extended precuneus cortex as stimulation sites of TPS. The frontal lobe is the most advanced part of brain development and controls movement, emotion, and mental activity. The parietal lobe also plays a role in memory retrieval.^42^ Other cognitive functions related to parietal lobe function can be impaired early in AD, such as attention, naming, or executive dysfunction.^43^ The precuneus is located in the posteromedial part of the parietal lobe. This area plays a central role in a wide range of integrated tasks, including visuospatial images, episodic memory retrieval, and self‐processing operations.^44^ Precuneus damage is related to the progression of AD.^45^ The different frequencies and stimulation sites of TPS in treating AD have not been explored. Therefore, future studies need to set blank or sham control groups and explore the optimal parameters and protocols for TPS treatment of AD.

For side effects, there are limited data reported in this systematic review. Adverse events in this review, including painless pressure sensations, pain, headache, worsening mood, jaw pain, nausea, and drowsiness, were reversible. In a study of TPS for lower limb spasticity in children with cerebral palsy, only mild skin redness (0.05%) at the electrode site was reported.^46^ The safety of TPS in the treatment of Parkinson's disease has been studied, and no adverse events have been observed.^47^ TPS is a low‐intensity transcranial electrical stimulation, and mild side effects similar to transcranial direct current stimulation (tDCS) may occur during treatment, such as itching, tingling, burning, and transient redness.^48^ Radjenovic et al.^18^ conducted a retrospective analysis of data on clinical TPS treatment in 101 patients with neurodegenerative diseases, more than 80% of whom reported no side effects during TPS and more than 60% of whom reported no adverse reactions after TPS. The most common adverse events were fatigue, dizziness, and pain. Notably, if side effects occurred, most patients reported these events after only 1 out of 10 sessions. Therefore, TPS in the treatment of AD may be safe, but it needs to be verified by high‐quality, large‐sample trials.

**FIGURE 2:**
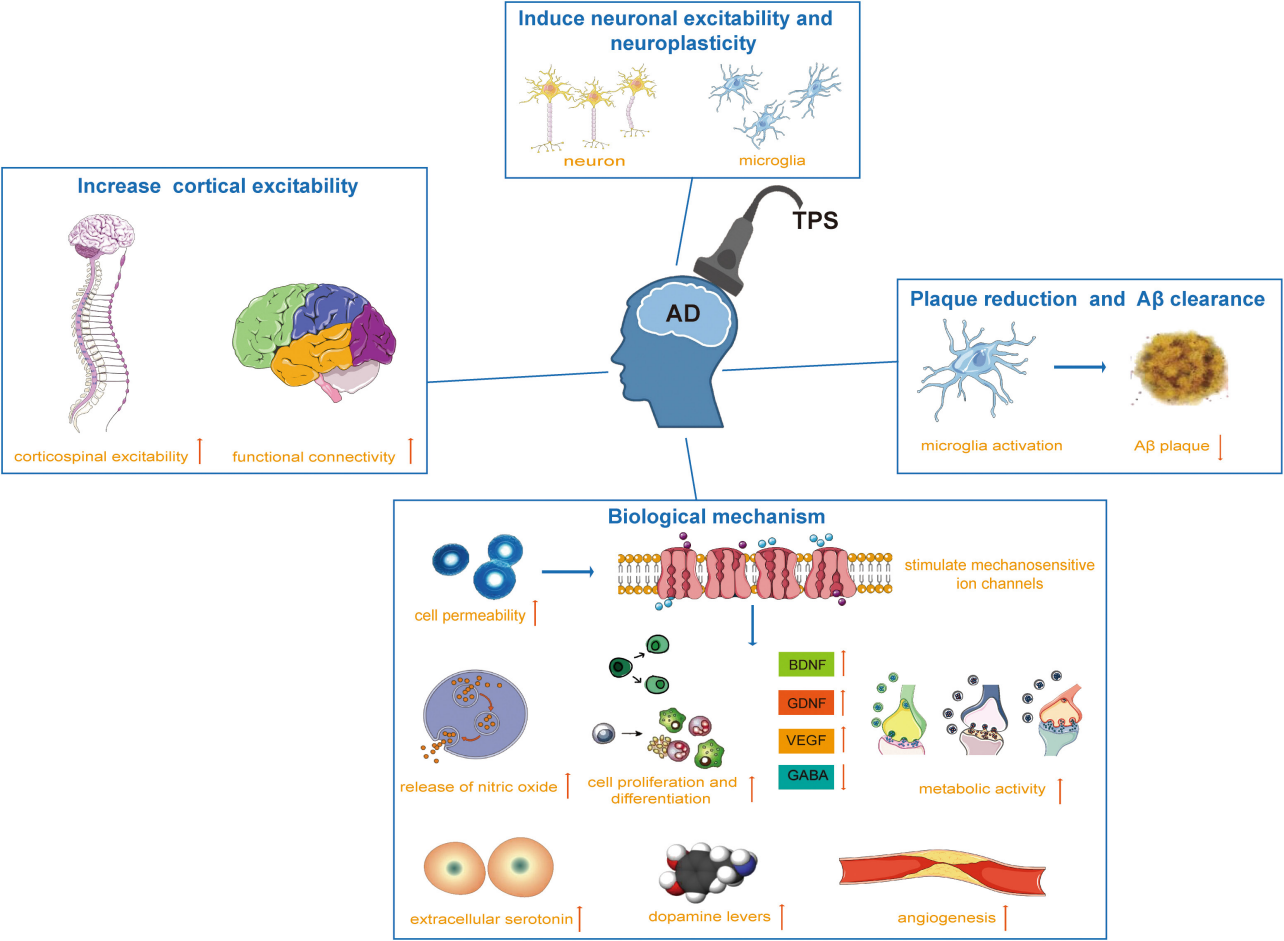
The mechanism of transcranial pulse stimulation in the treatment of Alzheimer's disease. AD, Alzheimer's disease; BDNF, brain‐derived neurotrophic factor; GABA, gamma‐aminobutyric acid; GDNF, glial cell line‑derived neurotrophic factor; TPS, transcranial pulse stimulation; VEGF, vascular growth factors.

Ultrasonic neuro‐regulation induces initial changes in cell permeability, leading to a series of changes in transmitters, humoral factors, and cell activity.^49^ Low‐intensity pulsed ultrasound reduces neuroinflammation by inhibiting the harmful overactivation of microglia.^50^ Hameroff et al.^51^ proposed that ultrasound directly affects cytoskeletal microtubules within neurons and glial cells. Figure 2 shows the mechanism of TPS in the treatment of AD. The biological mechanism of TPS is that cells convert mechanical TPS stimuli into biochemical responses, triggering some basic cell functions. TPS can stimulate mechanosensitive ion channels, promote cell proliferation and differentiation, stimulate brain‐derived neurotrophic factor (BDNF) and vascular growth factors (VEGF), improve cerebral blood flow, promote angiogenesis, and induce neuroplasticity.^52^, ^53^, ^54^, ^55^ Jaberzadeh et al.^56^ found that TPS with shorter intervals appears to have a greater impact on corticospinal excitability (CSE) with fewer side effects than tDCS with longer intervals of stimulation. tDCS modified neuronal excitability through tonic depolarization of the resting membrane potential, while TPS modified neuronal excitability through a combination of tonic and phasic effects.^56^ TPS has been shown to increase the power and connectivity of endogenous brain oscillations in a frequency‐specific manner^57^, ^58^ and interact with endogenous oscillatory activity to induce cortical excitability. It mainly increased the interhemispheric coherence of brain oscillatory activity in the frontotemporal region, thus enhancing the functional connectivity across neural networks.^39^, ^40^, ^59^ Matt et al.^60^ proposed that TPS increased functional and structural coupling within the left primary somatosensory cortex and adjacent sensorimotor areas more than a week after stimulation, suggesting that TPS induced neuroplasticity changes beyond the spatial and temporal stimulus environment. For cognitive function, TPS has been shown to have modest but specific cognitive effects, which can improve arithmetic processing on complex mathematical tasks, speech comprehension, and performance on attention‐shifting tasks in healthy subjects.^61^, ^62^, ^63^ Ultrasound therapy resulted in microglial activation with plaque reduction, Aβ clearance into microglial lysosomes, and improvements in spatial and recognition memory in an AD mouse model.^64^ Whole brain low‐intensity pulsed ultrasound therapy has been shown to significantly improve cognitive dysfunction in dementia mice by increasing the expression of endothelial oxide nitric synthase.^65^ In addition, Zarifka et al.^66^ found that TPS reversed Aβ‐induced impairment and improved Aβ‐induced memory in rat models of AD. For depression, decreased serum BDNF levels and decreased neuroplasticity are important pathophysiological mechanisms.^67^, ^68^ TPS increased BDNF expression and increased neuroplasticity, possibly thereby improving depressive symptoms in AD. However, the exact mechanism of action of TPS for AD is limited and requires more exploration.

This systematic review has some limitations. The main limitation is the inadequate methodology used in the included studies. The included studies did not set blank or sham control groups, so other factors may interfere with the authenticity of the results. Because TPS is a relatively new noninvasive transcranial ultrasound technique, there are only noncomparative studies. The lack of randomization, control, blindness, and follow‐up in these studies may lead to selection bias, measurement bias, and confounding bias. Second, three of the five included studies were conducted by the same working team, which may bias the results. Third, the small sample size included in this review may lead to an overall risk of bias or insufficient evidence. Four, we only retrieved data from English and Chinese databases, which may cause linguistic bias or limit data availability. Finally, due to the small number of included studies and insufficient data that could be pooled, we did not perform a meta‐analysis.

In conclusion, despite the lack of high‐quality randomized controlled trials, TPS has shown promise in improving cognitive performance and reducing depressive symptoms in patients with AD. Given the limited efficacy and side effects of existing drug therapies for AD, TPS may have potential as an adjunctive therapy for the management of AD.

## AUTHOR CONTRIBUTIONS

Conceptualization: CH, XXC. Funding Acquisition: CH. Formal Analysis: XXC. Investigation: CH. Writing–Original Draft Preparation: XXC, JHY, MZ, HM, CH. Writing–Review and Editing: all the authors. All the authors fulfill the ICMJE criteria for authorship.

## FUNDING INFORMATION

1·3·5 Project for Disciplines of excellence–Clinical Research Incubation Project, West China Hospital, Sichuan University (No. 2021HXFH063).

## CONFLICT OF INTEREST STATEMENT

The authors declare no financial relationships with any organizations that might have an interest in the submitted work and no other relationships or activities that could appear to have influenced the submitted work.

## Supporting information


Appendix S1

## Data Availability

Data are available upon reasonable request.

## References

[1] Alzheimer's Disease International . World Alzheimer Report 2018. The state of the art of dementia research: new frontiers. September, 2018. https://www.alzint.org/u/WorldAlzheimerReport2018.pdf (Accessed September 9, 2020).

[2] Burns A , Iliffe S . Alzheimer's disease. BMJ. 2009;338:b158. 19196745 10.1136/bmj.b158

[3] Revi M . Alzheimer's disease therapeutic approaches. Adv Exp Med Biol. 2020;1195:105‐116. 32468465 10.1007/978-3-030-32633-3_15

[4] Scheltens P , de Strooper B , Kivipelto M , et al. Alzheimer's disease. Lancet. 2021;397(10284):1577‐1590. 33667416 10.1016/S0140-6736(20)32205-4 PMC8354300

[5] Hodson R . Alzheimer's disease. Nature. 2018;559(7715):S1. 30046078 10.1038/d41586-018-05717-6

[6] Schott JM , Aisen PS , Cummings JL , Howard RJ , Fox NC . Unsuccessful trials of therapies for Alzheimer's disease. Lancet. 2019;393(10166):29. 10.1016/S0140-6736(18)31896-8 30614456

[7] Breijyeh Z , Karaman R . Comprehensive review on Alzheimer's disease: causes and treatment. Molecules. 2020;25(24):5789. 33302541 10.3390/molecules25245789 PMC7764106

[8] Shafqat S . Alzheimer disease therapeutics: perspectives from the developing world. J Alzheimers Dis. 2008;15(2):285‐287. 18953114 10.3233/jad-2008-15211

[9] Tatulian SA . Challenges and hopes for Alzheimer's disease. Drug Discov Today. 2022;27(4):1027‐1043. 35121174 10.1016/j.drudis.2022.01.016

[10] Chu CS , Li CT , Brunoni AR , et al. Cognitive effects and acceptability of non‐invasive brain stimulation on Alzheimer's disease and mild cognitive impairment: a component network meta‐analysis. J Neurol Neurosurg Psychiatry. 2021;92(2):195‐203. 33115936 10.1136/jnnp-2020-323870 PMC7841477

[11] Gu L , Xu H , Qian F . Effects of non‐invasive brain stimulation on Alzheimer's disease. J Prev Alzheimers Dis. 2022;9(3):410‐424. 35841242 10.14283/jpad.2022.40

[12] Menardi A , Rossi S , Koch G , et al. Toward noninvasive brain stimulation 2.0 in Alzheimer's disease. Ageing Res Rev. 2022;75:101555. 34973457 10.1016/j.arr.2021.101555 PMC8858588

[13] Teselink J , Bawa KK , Koo GK , et al. Efficacy of non‐invasive brain stimulation on global cognition and neuropsychiatric symptoms in Alzheimer's disease and mild cognitive impairment: a meta‐analysis and systematic review. Ageing Res Rev. 2021;72:101499. 34700007 10.1016/j.arr.2021.101499

[14] Minjoli S , Saturnino GB , Blicher JU , et al. The impact of large structural brain changes in chronic stroke patients on the electric field caused by transcranial brain stimulation. NeuroImage Clin. 2017;15:106‐117. 28516033 10.1016/j.nicl.2017.04.014 PMC5426045

[15] Deng ZD , Lisanby SH , Peterchev AV . Coil design considerations for deep transcranial magnetic stimulation. Clin Neurophysiol. 2014;125(6):1202‐1212. 24411523 10.1016/j.clinph.2013.11.038 PMC4020988

[16] Szabo TL . Diagnostic Ultrasound Imaging: Inside Out. Elsevier Academic Press; 2014.

[17] Truong DQ , Thomas C , Hampstead BM , Datta A . Comparison of transcranial focused ultrasound and transcranial pulse stimulation for neuromodulation: a computational study. Neuromodulation. 2022;25(4):606‐613. 35125300 10.1016/j.neurom.2021.12.012

[18] Radjenovic S , Dörl G , Gaal M , Beisteiner R . Safety of clinical ultrasound neuromodulation. Brain Sci. 2022;12(10):1277. 36291211 10.3390/brainsci12101277 PMC9599299

[19] Legon W , Sato TF , Opitz A , et al. Transcranial focused ultrasound modulates the activity of primary somatosensory cortex in humans. Nat Neurosci. 2014;17(2):322‐329. 24413698 10.1038/nn.3620

[20] Legon W , Ai L , Bansal P , Mueller JK . Neuromodulation with single‐element transcranial focused ultrasound in human thalamus. Hum Brain Mapp. 2018;39(5):1995‐2006. 29380485 10.1002/hbm.23981 PMC6866487

[21] Datta A , Dmochowski JP , Guleyupoglu B , Bikson M , Fregni F . Cranial electrotherapy stimulation and transcranial pulsed current stimulation: a computer based high‐resolution modeling study. Neuroimage. 2013;65:280‐287. 23041337 10.1016/j.neuroimage.2012.09.062

[22] Collins MN , Legon W , Mesce KA . The inhibitory thermal effects of focused ultrasound on an identified, Single Motoneuron. eNeuro. 2021;8(2):ENEURO.0514‐ENEU20.2021. 10.1523/ENEURO.0514-20.2021 PMC8174046 33853851

[23] Darrow DP , O'Brien P , Richner TJ , Netoff TI , Ebbini ES . Reversible neuroinhibition by focused ultrasound is mediated by a thermal mechanism. Brain Stimul. 2019;12(6):1439‐1447. 31377096 10.1016/j.brs.2019.07.015 PMC6851480

[24] Mueller JK , Ai L , Bansal P , Legon W . Numerical evaluation of the skull for human neuromodulation with transcranial focused ultrasound. J Neural Eng. 2017;14(6):066012. 28777075 10.1088/1741-2552/aa843e

[25] Beisteiner R , Matt E , Fan C , et al. Transcranial Pulse Stimulation with Ultrasound in Alzheimer's Disease‐A New Navigated Focal Brain Therapy. Adv Sci (Weinh). 2019;7(3):1902583. 32042569 10.1002/advs.201902583 PMC7001626

[26] Cont C , Stute N , Galli A , et al. Retrospective real‐world pilot data on transcranial pulse stimulation in mild to severe Alzheimer's patients. Front Neurol. 2022;13:948204. 36188380 10.3389/fneur.2022.948204 PMC9515314

[27] Dörl G , Matt E , Beisteiner R . Functional specificity of TPS brain stimulation effects in patients with Alzheimer's disease: a follow‐up fMRI analysis. Neurol Ther. 2022;11(3):1391‐1398. 35633496 10.1007/s40120-022-00362-8 PMC9338196

[28] Matt E , Dörl G , Beisteiner R . Transcranial pulse stimulation (TPS) improves depression in AD patients on state‐of‐the‐art treatment. Alzheimers Dement (N Y). 2022;8(1):e12245. 35169611 10.1002/trc2.12245 PMC8829892

[29] Popescu T , Pernet C , Beisteiner R . Transcranial ultrasound pulse stimulation reduces cortical atrophy in Alzheimer's patients: a follow‐up study. Alzheimers Dement (N Y). 2021;7(1):e12121. 33681449 10.1002/trc2.12121 PMC7906128

[30] Page MJ , McKenzie JE , Bossuyt PM , et al. The PRISMA 2020 statement: an updated guideline for reporting systematic reviews. BMJ. 2021;372:n71. 33782057 10.1136/bmj.n71 PMC8005924

[31] Ehrensperger MM , Berres M , Taylor KI , Monsch AU . Early detection of Alzheimer's disease with a total score of the German CERAD. J Int Neuropsychol Soc. 2010;16(5):910‐920. 20682088 10.1017/S1355617710000822

[32] Cano SJ , Posner HB , Moline ML , et al. The ADAS‐cog in Alzheimer's disease clinical trials: psychometric evaluation of the sum and its parts. J Neurol Neurosurg Psychiatry. 2010;81(12):1363‐1368. 20881017 10.1136/jnnp.2009.204008

[33] Slim K , Nini E , Forestier D , Kwiatkowski F , Panis Y , Chipponi J . Methodological index for non‐randomized studies (minors): development and validation of a new instrument. ANZ J Surg. 2003;73(9):712‐716. 12956787 10.1046/j.1445-2197.2003.02748.x

[34] Kueper JK , Speechley M , Montero‐Odasso M . The Alzheimer's disease assessment scale‐cognitive subscale (ADAS‐cog): modifications and responsiveness in pre‐dementia populations. A narrative review. J Alzheimers Dis. 2018;63(2):423‐444. 29660938 10.3233/JAD-170991 PMC5929311

[35] Tombaugh TN , McIntyre NJ . The mini‐mental state examination: a comprehensive review. J Am Geriatr Soc. 1992;40(9):922‐935. 1512391 10.1111/j.1532-5415.1992.tb01992.x

[36] Trzepacz PT , Hochstetler H , Wang S , Walker B , Saykin AJ . Relationship between the Montreal cognitive assessment and mini‐mental state examination for assessment of mild cognitive impairment in older adults. BMC Geriatr. 2015;15:107. 26346644 10.1186/s12877-015-0103-3 PMC4562190

[37] Dunlop K , Hanlon CA , Downar J . Noninvasive brain stimulation treatments for addiction and major depression. Ann N Y Acad Sci. 2017;1394(1):31‐54. 26849183 10.1111/nyas.12985 PMC5434820

[38] Fitzgerald PB . Transcranial pulsed current stimulation: a new way forward? Clin Neurophysiol. 2014;125(2):217‐219. 24210514 10.1016/j.clinph.2013.10.009

[39] Castillo Saavedra L , Morales‐Quezada L , Doruk D , et al. QEEG indexed frontal connectivity effects of transcranial pulsed current stimulation (tPCS): a sham‐controlled mechanistic trial. Neurosci Lett. 2014;577:61‐65. 24937270 10.1016/j.neulet.2014.06.021

[40] Morales‐Quezada L , Saavedra LC , Rozisky J , Hadlington L , Fregni F . Intensity‐dependent effects of transcranial pulsed current stimulation on interhemispheric connectivity: a high‐resolution qEEG, sham‐controlled study. Neuroreport. 2014;25(13):1054‐1058. 25055142 10.1097/WNR.0000000000000228

[41] Morales‐Quezada L , Castillo‐Saavedra L , Cosmo C , et al. Optimal random frequency range in transcranial pulsed current stimulation indexed by quantitative electroencephalography. Neuroreport. 2015;26(13):747‐752. 26154494 10.1097/WNR.0000000000000415

[42] Cabeza R , Ciaramelli E , Olson IR , Moscovitch M . The parietal cortex and episodic memory: an attentional account. Nat Rev Neurosci. 2008;9(8):613‐625. 18641668 10.1038/nrn2459 PMC2692883

[43] Lindeboom J , Weinstein H . Neuropsychology of cognitive ageing, minimal cognitive impairment, Alzheimer's disease, and vascular cognitive impairment. Eur J Pharmacol. 2004;490(1–3):83‐86. 15094075 10.1016/j.ejphar.2004.02.046

[44] Cavanna AE , Trimble MR . The precuneus: a review of its functional anatomy and behavioural correlates. Brain. 2006;129(Pt 3):564‐583. 16399806 10.1093/brain/awl004

[45] Li K , Wang S , Luo X , Zeng Q , Jiaerken Y , Xu X , et al. Progressive memory circuit impairments along with Alzheimer's disease neuropathology spread: evidence from in vivo neuroimaging. Cerebral Cortex. 2020;30(11):5863–73. 32537637 10.1093/cercor/bhaa162

[46] Liu Z , Dong S , Zhong S , et al. The effect of combined transcranial pulsed current stimulation and transcutaneous electrical nerve stimulation on lower limb spasticity in children with spastic cerebral palsy: a randomized and controlled clinical study. BMC Pediatr. 2021;21(1):141. 33761932 10.1186/s12887-021-02615-1 PMC7989146

[47] Alon G , Yungher DA , Shulman LM , Rogers MW . Safety and immediate effect of noninvasive transcranial pulsed current stimulation on gait and balance in Parkinson disease. Neurorehabil Neural Repair. 2012;26(9):1089‐1095. 22581566 10.1177/1545968312448233

[48] Brunoni AR , Amadera J , Berbel B , Volz MS , Rizzerio BG , Fregni F . A systematic review on reporting and assessment of adverse effects associated with transcranial direct current stimulation. Int J Neuropsychopharmacol. 2011;14(8):1133‐1145. 21320389 10.1017/S1461145710001690

[49] Babakhanian M , Yang L , Nowroozi B , et al. Effects of low intensity focused ultrasound on liposomes Containing Channel proteins. Sci Rep. 2018;8(1):17250. 30467339 10.1038/s41598-018-35486-1 PMC6250712

[50] Beisteiner R , Lozano AM . Transcranial ultrasound innovations ready for broad clinical application. Adv Sci (Weinh). 2020;7(23):2002026. 33304757 10.1002/advs.202002026 PMC7709976

[51] Hameroff S , Trakas M , Duffield C , et al. Transcranial ultrasound (TUS) effects on mental states: a pilot study. Brain Stimul. 2013;6(3):409‐415. 22664271 10.1016/j.brs.2012.05.002

[52] Hatanaka K , Ito K , Shindo T , et al. Molecular mechanisms of the angiogenic effects of low‐energy shock wave therapy: roles of mechanotransduction. Am J Physiol Cell Physiol. 2016;311(3):C378‐C385. 27413171 10.1152/ajpcell.00152.2016

[53] Wang B , Ning H , Reed‐Maldonado AB , et al. Low‐intensity extracorporeal shock wave therapy enhances brain‐derived neurotrophic Factor expression through PERK/ATF4 signaling pathway. Int J Mol Sci. 2017;18(2):433. 28212323 10.3390/ijms18020433 PMC5343967

[54] Zhang J , Kang N , Yu X , Ma Y , Pang X . Radial extracorporeal shock wave therapy enhances the proliferation and differentiation of neural stem cells by notch, PI3K/AKT, and Wnt/β‐catenin signaling. Sci Rep. 2017;7(1):15321. 29127399 10.1038/s41598-017-15662-5 PMC5681501

[55] Cheung T , Li TMH , Ho YS , et al. Effects of transcranial pulse stimulation (TPS) on adults with symptoms of depression‐a pilot randomized controlled trial. Int J Environ Res Public Health. 2023;20(3):2333. 36767702 10.3390/ijerph20032333 PMC9915638

[56] Jaberzadeh S , Bastani A , Zoghi M . Anodal transcranial pulsed current stimulation: a novel technique to enhance corticospinal excitability. Clin Neurophysiol. 2014;125(2):344‐351. 24074626 10.1016/j.clinph.2013.08.025

[57] Thibaut A , Russo C , Morales‐Quezada L , et al. Neural signature of tDCS, tPCS and their combination: comparing the effects on neural plasticity. Neurosci Lett. 2017;637:207‐214. 27765610 10.1016/j.neulet.2016.10.026 PMC5541936

[58] Singh A , Trapp NT , De Corte B , et al. Cerebellar theta frequency transcranial pulsed stimulation increases frontal theta oscillations in patients with schizophrenia. Cerebellum. 2019;18(3):489‐499. 30825131 10.1007/s12311-019-01013-9 PMC6818969

[59] Vasquez A , Malavera A , Doruk D , et al. Duration dependent effects of transcranial pulsed current stimulation (tPCS) indexed by electroencephalography. Neuromodulation. 2016;19(7):679‐688. 27400423 10.1111/ner.12457

[60] Matt E , Kaindl L , Tenk S , et al. First evidence of long‐term effects of transcranial pulse stimulation (TPS) on the human brain. J Transl Med. 2022;20(1):26. 35033118 10.1186/s12967-021-03222-5 PMC8760674

[61] Morales‐Quezada L , Cosmo C , Carvalho S , et al. Cognitive effects and autonomic responses to transcranial pulsed current stimulation. Exp Brain Res. 2015;233(3):701‐709. 25479736 10.1007/s00221-014-4147-y

[62] Morales‐Quezada L , Leite J , Carvalho S , Castillo‐Saavedra L , Cosmo C , Fregni F . Behavioral effects of transcranial pulsed current stimulation (tPCS): speed‐accuracy tradeoff in attention switching task. Neurosci Res. 2016;109:48‐53. 26851768 10.1016/j.neures.2016.01.009

[63] Ruhnau P , Rufener KS , Heinze HJ , Zaehle T . Pulsed transcranial electric brain stimulation enhances speech comprehension. Brain Stimul. 2020;13(5):1402‐1411. 32735988 10.1016/j.brs.2020.07.011

[64] Leinenga G , Götz J . Scanning ultrasound removes amyloid‐β and restores memory in an Alzheimer's disease mouse model. Sci Transl Med. 2015;7(278):278ra33. 10.1126/scitranslmed.aaa2512 25761889

[65] Eguchi K , Shindo T , Ito K , et al. Whole‐brain low‐intensity pulsed ultrasound therapy markedly improves cognitive dysfunctions in mouse models of dementia ‐ crucial roles of endothelial nitric oxide synthase. Brain Stimul. 2018;11(5):959‐973. 29857968 10.1016/j.brs.2018.05.012

[66] Zarifkar AH , Zarifkar A , Nami M , Rafati A , Aligholi H , Vafaee F . Ameliorative effects of different transcranial electrical stimulation paradigms on the novel object recognition task in a rat model of Alzheimer disease. Galen Med J. 2019;8:e1440. 34466513 10.31661/gmj.v8i0.1440 PMC8344121

[67] Kraus C , Castrén E , Kasper S , Lanzenberger R . Serotonin and neuroplasticity ‐ links between molecular, functional and structural pathophysiology in depression. Neurosci Biobehav Rev. 2017;77:317‐326. 28342763 10.1016/j.neubiorev.2017.03.007

[68] Colucci‐D'Amato L , Speranza L , Volpicelli F . Neurotrophic factor BDNF, physiological functions and therapeutic potential in depression, neurodegeneration and brain cancer. Int J Mol Sci. 2020;21(20):7777. 33096634 10.3390/ijms21207777 PMC7589016

