# Supplementary material for: Transcranial pulse stimulation in Alzheimer's disease
Source: CNS Neurosci Ther. 2023 Jul 20;30(2):e14372. doi: 10.1111/cns.14372 (PMC10848065; doi:10.1111/cns.14372)
Supplement: Supplementary file 1 — Appendix S1 [file CNS-30-e14372-s001.pdf]

Pubmed

Search	Query	Results	Time
#3	Search: ((Alzheimer disease[MeSH Terms]) OR (Alzheimer)) AND (((transcranial pulsed current[Title/Abstract]) OR (transcranial ultrasound[Title/Abstract])) OR (transcranial pulse[Title/Abstract]))	24	20:26:46
#2	Search: ((transcranial pulsed current[Title/Abstract]) OR (transcranial ultrasound[Title/Abstract])) OR (transcranial pulse[Title/Abstract])	650	20:26:36
#1	Search: (Alzheimer disease[MeSH Terms]) OR (Alzheimer)	211,383	20:23:31

Search	Actions	Details	Query	Results	Time
#3	•••	>	Search: **((Alzheimer disease[MeSH Terms]) OR (Alzheimer)) AND (((transcranial pulsed current[Title/Abstract]) OR (transcranial ultrasound[Title/Abstract])) OR (transcranial pulse[Title/Abstract]))**	24	20:26:46
#2	•••	>	Search: **((transcranial pulsed current[Title/Abstract]) OR (transcranial ultrasound[Title/Abstract])) OR (transcranial pulse[Title/Abstract])**	650	20:26:36
#1	•••	>	Search: **(Alzheimer disease[MeSH Terms]) OR (Alzheimer)**	211,383	20:23:31

Showing 1 to 3 of 3 entries

Embase via Ovid

1
exp Alzheimer disease/
245904
2
Alzheimer.mp.
255338
3
1 or 2
255338
4
transcranial pulse.mp.
44
5
transcranial pulsed current.mp.
51
6
transcranial ultrasound.mp.
1006

7
4 or 5 or 6
1097
8
3 and 7
46

# ▲	Searches	Results	Type	Actions	Annotations
1	exp Alzheimer disease/	245904	Advanced	Display Results More	
2	Alzheimer.mp.	255338	Advanced	Display Results More	
3	1 or 2	255338	Advanced	Display Results More	
4	transcranial pulse.mp.	44	Advanced	Display Results More	
5	transcranial pulsed current.mp.	51	Advanced	Display Results More	
6	transcranial ultrasound.mp.	1006	Advanced	Display Results More	
7	4 or 5 or 6	1097	Advanced	Display Results More	
8	3 and 7	46	Advanced	Display Results More	

Web of Science (1900-present)

1
Topic= (“Alzheimer's disease” OR "Alzheimer")
333490
2
Topic= (“transcranial pulse” OR “transcranial pulsed current” OR “transcranial ultrasound”)
13680
3
1 AND 2
271

0/3 Assemble search queries Export

3	#1 AND #2	271	Add to a search query
2	((TS=(transcranial pulse)) OR TS=(transcranial pulsed current)) OR TS=(transcranial ultrasound)	13,680	Add to a search query
1	(TS=(Alzheimer's disease)) OR TS=(Alzheimer)	333,490	Add to a search query

Cochrane Library

ID Search Hits
#1 MeSH descriptor: [Alzheimer disease] explode all trees 4454

#2 (Alzheimer's disease): ti,ab,kw 12480
#3 (Alzheimer): ti,ab,kw 13103
#4 #2 OR #3
#5 (transcranial pulse): ti,ab,kw 708
#6 (transcranial pulsed current): ti,ab,kw 47
#7 (transcranial ultrasound): ti,ab,kw 495
#8 #1 OR #4 13103
#9 #5 OR #6 OR #7 1202
#10 #8 AND #9 23

View fewer lines | Print search history

#1	MeSH descriptor: [Alzheimer Disease] explode all trees	MeSH		4454
#2	(Alzheimer's disease):ti,ab,kw	S	Limits	12480
#3	(Alzheimer):ti,ab,kw	S	Limits	13103
#4	#2 OR #3		Limits	13103
#5	(transcranial pulse):ti,ab,kw	S	Limits	708
#6	(transcranial pulsed current):ti,ab,kw	S	Limits	47
#7	(transcranial ultrasound):ti,ab,kw	S	Limits	495
#8	#1 OR #4		Limits	13103
#9	#5 OR #6 OR #7		Limits	1202
#10	#8 AND #9		Limits	23

Clear all
Highlight orphan lines

CNKI (Chinese database)
(篇关摘=阿尔兹海默病) AND（篇关摘=经颅脉冲 OR 篇关摘=经颅超声）
0

VIP (Chinese database)
(题名或关键词=阿尔兹海默病）AND（题名或关键词=经颅脉冲 OR 题名或关键词=经颅超声）
0

WANFANG (Chinese database)
题名或关键词：阿尔兹海默病 and（经颅脉冲 or 经颅超声）
0
